# Development and clinical application of biological dosimetry technology: insights from the HICARE/IAEA 2025 international training course

**DOI:** 10.1093/jrr/rrag003

**Published:** 2026-02-24

**Authors:** Wanwisa Sudprasert, Oleg Belyakov, Satoshi Tashiro

**Affiliations:** Department of Applied Radiation and Isotopes, Faculty of Science, Kasetsart University, 50 Ngam Wong Wan Road, Chatuchak, 10900 Bangkok, Thailand; Department of Nuclear Sciences and Applications, International Atomic Energy Agency, Vienna International Centre, PO Box 100, 1400 Vienna, Austria; Hiroshima International Council for Health Care of the Radiation-Exposed, 10-52, Motomachi, Nakaku, 730-8511 Hiroshima, Japan; Department of Cellular Biology, Research Institute for Radiation Biology and Medicine, Hiroshima University, 1-2-3 Kasumi, Minamiku, 734-8553 Hiroshima, Japan

**Keywords:** biological dosimetry, cytogenetic assays, internal dosimetry, radiological emergency preparedness, radiation risk assessment, capacity building

## Abstract

The HICARE/IAEA 2025 International Training Course on Biological and Internal Dosimetry, held at Hiroshima University from 10 to 14 February 2025, convened 20 participants from nine countries— Brazil, Indonesia, Japan, Korea, the Philippines, Singapore, Thailand, the United States, and Vietnam—and was jointly organized by the Hiroshima International Council for Health Care of the Radiation-Exposed (HICARE) and the International Atomic Energy Agency (IAEA). With guidance from 13 expert lecturers, the course aimed to enhance global capacity in radiation dose assessment and clinical application of biodosimetry. The program, led by Prof. Satoshi Tashiro (HICARE) and Dr. Oleg Belyakov (IAEA), included lectures on cytogenetic assays, molecular biomarkers, and internal dosimetry using biokinetic modeling and bioassay data. Key themes emphasized the integration of biodosimetry into clinical decision-making, emergency preparedness, and occupational health monitoring. Discussions highlighted the expanding role of biodosimetry in complementing internal dose assessments for patients undergoing diagnostic imaging procedures—such as CT scans and nuclear medicine—as well as for individuals affected by radiological accidents. These sessions underscored the need for harmonized protocols, reliable dose reconstruction methods, and cross-institutional networks to support accurate and timely biodosimetric evaluations in both clinical and emergency settings. The workshop not only had advanced technical competencies but also positioned biodosimetry as a critical tool in global radiological protection strategies. Strong participant engagement and interest in future events reflected the course’s impact on strengthening international cooperation and translating research into practice.

## INTRODUCTION

Biological dosimetry, or biodosimetry, encompasses a suite of cytogenetic and molecular methods used to estimate absorbed radiation doses by examining biological markers in exposed individuals. These measurements are essential for guiding clinical interventions following radiotherapy, assessing health risks in occupational settings, and coordinating emergency responses to radiological incidents [1, 2]. While established methods—such as the dicentric chromosome assay—remain foundational, the increasing relevance of low-dose and internal exposures demands continuous skill development and methodological standardization [1].

Internal dosimetry complements biodosimetry by focusing on the quantification of radionuclide intake and distribution within the human body. Through a combination of *in vivo* measurements (e.g. whole-body counting) and bioassay analyses (e.g. urine and fecal samples), practitioners use biokinetic and dosimetric models to reconstruct organ-specific dose estimates [3]. Such integration of physical measurements with biological endpoints offers a comprehensive understanding of both the physical deposition of radioactive materials and their subsequent biological effects, enhancing dose assessment accuracy for medical, occupational, and emergency contexts [4, 5].

Recognizing these needs, Hiroshima International Council for Health Care of the Radiation-Exposed (HICARE) and the International Atomic Energy Agency (IAEA) launched an international workshop to bridge the gap between theoretical understanding and practical application in biodosimetry. Building on previous collaborations, the two organizations had earlier conducted two major training events in Hiroshima. The *BIODOSE-21* workshop, held in June 2013, aimed to strengthen knowledge of both classical and emerging biodosimetry techniques. It brought together 25 participants, including 11 invited lecturers from across the Asia-Pacific region, under the auspices of IAEA CRP E35008. A second workshop, *Biological and Internal Dosimetry: Recent Advances and Clinical Applications*, held in February 2020 as part of the IAEA Technical Cooperation project RAS9087, gathered 19 participants from various countries to explore dosimetry strategies relevant to accidental, environmental, and medical exposures [6]. Lessons learned from these two events helped shape the structure and focus of the 2025 workshop.

**Fig. 1:**
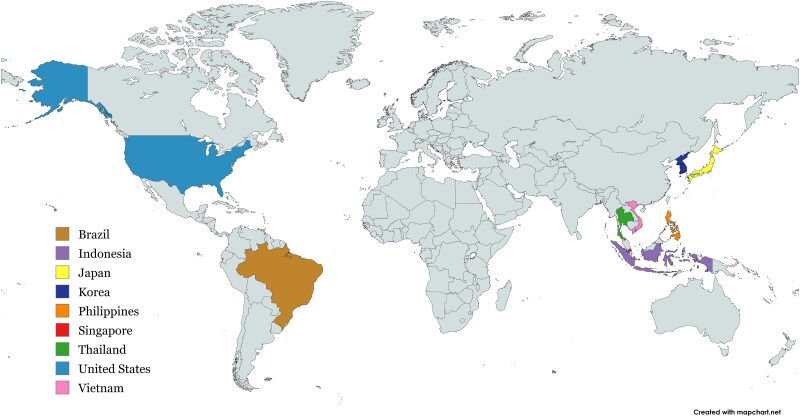
Geographic distribution of participants in the HICARE/IAEA 2025 International Training Course, representing nine countries across Asia and the Americas. Map adapted from MapChart (https://mapchart.net), licensed under CC BY-SA 4.0.

The 2025 workshop, titled *Biological and Internal Dosimetry: Development and Clinical Application of Biological Dosimetry Technology*, was directed by Prof. Satoshi Tashiro of HICARE and Dr. Oleg Belyakov of the IAEA, and was held at Hiroshima University from 10 to 14 February 2025. It marked the third collaborative training meeting between HICARE and the IAEA, further solidifying a decade-long partnership aimed at strengthening global biodosimetry capacity. The workshop welcomed 20 participants from nine countries: Brazil, Indonesia, Japan, Korea, the Philippines, Singapore, Thailand, the United States, and Vietnam (Fig. 1). Its primary objective was to enhance participants’ capabilities in applying both established and innovative biological dosimetry techniques—such as dicentric chromosome analysis, γ-H2AX foci enumeration, and molecular biomarker assays—alongside internal dosimetry approaches for reconstructing organ-specific doses based on bioassay and monitoring data. By combining theoretical lectures with participant presentations, the program enabled attendees to gain technical proficiency, critically evaluate the strengths and limitations of each method, and explore the translation of biodosimetry into clinical applications. This comprehensive curriculum ensured that participants were well-equipped to implement biodosimetry protocols in their home institutions and contribute to collaborative research efforts.

Compared with the previous HICARE/IAEA training courses (2013 and 2020), the 2025 program demonstrated continued progress in international engagement, methodological advancement, and emergency-response capacity. While the number of participating countries in 2025 (nine countries) was comparable to that of the 2020 workshop, the program reflected an expanded geographical balance and strengthened regional collaboration, including broader representation from East and Southeast Asia.

In addition, the workshop highlighted significant advances in assay sensitivity and throughput, particularly for low-dose exposure assessment. The introduction of γ-H2AX high-throughput scoring, automated metaphase detection, and the integration of biokinetic modeling for internal dose reconstruction has enhanced the ability to detect and quantify lower radiation doses compared with earlier training periods. These developments reduced analysis time by ~30–50% relative to fully manual cytogenetic approaches and increased the number of biological samples that can be processed in emergency situations—from several tens to more than one hundred samples per week—thereby strengthening preparedness for large-scale radiological events and low-dose exposure scenarios.

### Program highlights

#### Day 1 (Monday): IAEA biological dosimetry research program

The workshop opened with remarks by Dr. Belyakov (IAEA) and Prof. Tashiro (Hiroshima University), highlighting the importance of international collaboration and the growing role of biodosimetry in clinical and emergency preparedness. Their introduction framed the workshop as a platform for exchanging practical experience and strengthening regional and global capacity.

Dr. Belyakov presented an overview of the IAEA Biodosimetry Research Program, emphasizing advances achieved through two Coordinated Research Projects (CRP E35008 and E35010). These CRPs strengthened capacity across participating countries by validating classical cytogenetic assays and promoting newer molecular approaches such as γH2AX focus analysis and gene expression profiling. He also announced a forthcoming CRP on automation and AI-based biodosimetry (planned for 2026), noting that Biological Dosimetry Model Laboratory (BDML) in Seibersdorf now supports assay standardization and training worldwide. Key progress areas included improved assay reproducibility, faster turnaround times through partial automation, and enhanced readiness for radiological emergencies [7].

Prof. Tashiro followed with an overview of clinical applications. He summarized how cytogenetic biomarkers—including dicentrics, rings, translocations, and γH2AX foci—are used to assess medical radiation exposures [8], supported by evidence from CT imaging studies [9, 10] and radioiodine therapy [11]. He also presented longitudinal cases showing the persistence of chromosomal abnormalities after internal radionuclide exposure, reinforcing the importance of biodosimetry in personalized radiotherapy planning and long-term patient follow-up [12].

A lecture delivered by Dr. Belyakov on behalf of Dr. Sergey Shinkarev (FMBC) highlighted practical lessons from Chernobyl and Fukushima, focusing on rapid thyroid monitoring for I-131. The session summarized improvements in field measurement methods, calibration practices, and uncertainty reduction strategies—demonstrating clear progress in mass-screening logistics and early dose reconstruction for first responders and the public [13, 14].

Dr. Osamu Kurihara (QST) provided an overview of internal dosimetry, explaining the use of biokinetic models—such as the Human Respiratory Tract Model (HRTM) and Human Alimentary Tract Model (HATM) [15, 16]—and bioassay data in organ dose reconstruction. A case study from the 2017 JAEA plutonium incident illustrated current capabilities in intake estimation and medical intervention [17]. Key advances included the integration of multiple measurement types and refinement of dose assessment models for real-world exposure scenarios.

Prof. Kimio Tanaka (Hiroshima University) summarized long-term low-dose-rate irradiation studies, demonstrating clear dose–response relationships in chromosomal aberrations and life-span reduction [18, 19]. His work supports the use of stable translocations as long-term biomarkers and highlights that cumulative dose—rather than dose rate alone—plays a decisive role in long-term biological outcomes [20]. He also addressed challenges in retrospective biodosimetry and emphasized the need for improved correction models for age and individual variability.

The first day concluded with a consolidated understanding of both classical and modern biodosimetric approaches, with emphasis on (i) progress achieved through IAEA research networks, (ii) advances in molecular assays and internal dose reconstruction, and (iii) the expanding clinical utility of biodosimetry. These foundations prepared participants for deeper practical and technical sessions in the following days.

#### Day 2 (Tuesday): internal dose assessment and clinical protection

Dr. Shinkarev’s lecture (delivered by Dr. Belyakov) provided a comparative overview of internal thyroid dose assessment from Chernobyl and Fukushima. He highlighted how biokinetic models and personal intake histories allow reconstruction of I-131 thyroid doses [21], noting that Chernobyl exposures—dominated by ingestion—reached tens of grays in children [22], whereas Fukushima inhalation exposures remained largely below a few hundred mGy [23]. He stressed that early direct thyroid measurements dramatically improve accuracy, as demonstrated by the >400 000 measurements collected after Chernobyl, compared to limited datasets in Fukushima [13]. Key takeaways included the critical role of rapid monitoring, age- and behavior-specific dose modifiers, and the need for well-prepared large-scale thyroid screening systems in emergency scenarios.

Prof. Tomisato Miura (Hirosaki University) presented occupational exposure findings among orthopedic surgeons using fluoroscopy. Using DCA and translocation analysis, his team reported significantly elevated chromosomal aberrations compared with controls [24, 25], reflecting the cumulative impact of chronic low-dose scatter radiation. Although physical dose estimates remained below deterministic thresholds, the frequency of stable aberrations suggested potential long-term cancer risks. He emphasized the need for stronger radiation protection—improved shielding, routine monitoring, and better awareness of scatter radiation hazards in medical practice.

In the afternoon, Dr. Nobuki Imano (Hiroshima University) provided an overview of modern radiation therapy concepts and technologies. He explained radiobiological principles underlying fractionation [26], and described major advances such as IMRT/VMAT, stereotactic techniques, and image-guided radiotherapy. He also reviewed clinical applications of radioisotope therapy (e.g. ^131^I, ^177^Lu agents) and emphasized both therapeutic benefits and risks of acute and late toxicities, including the possibility of secondary malignancies [27]. His session underscored the need for ongoing optimization to maximize tumor control while minimizing radiation-induced complications.

Dr. Kanya Hamasaki (RERF) summarized extensive chromosomal studies in A-bomb survivors that underpin biological dosimetry. Using both conventional Giemsa staining and fluorescence *in situ* hybridization (FISH), consistent dose–response relationships were demonstrated for chromosomal aberrations, with stable translocations persisting as reliable long-term biomarkers of radiation exposure [28, 29]. Comparisons among FISH-based dose estimates, the DS02R1 physical dosimetry system, and electron spin resonance (ESR) measurements in teeth showed high concordance, supporting the validity of chromosome-based biodosimetry. Importantly, studies of individuals exposed *in utero* revealed no persistent increase in chromosomal aberrations, despite clear dose–response relationships in their mothers, a finding further supported by experimental animal models [30, 31]. Together, these results reinforce the complementary roles of epidemiological and cytogenetic approaches in retrospective dose assessment and in understanding the long-term biological consequences of radiation exposure.

The final session by Dr. Sakae Kinase (JAEA) reviewed key practices in internal monitoring of radiation workers. He summarized in vivo and in vitro assessment methods, calibration requirements, and the use of phantoms, minimum detectable doses (MDDs) and derived recording levels (DRLs) in designing monitoring programs aligned with ICRP and IAEA standards [32–34]. Advances such as voxel-phantom–based calibration and Monte Carlo simulations were highlighted as tools that improve accuracy and reduce uncertainty [35]. Case examples illustrated the importance of confirmatory monitoring during unusual workplace conditions and the value of updated bioassay protocols and real-time surveillance systems.

Day 2 provided participants with an integrated view of internal dose assessment in emergencies and occupational settings, supported by real-world evidence from nuclear accidents and clinical environments. The sessions highlighted improved methodologies, practical challenges, and the essential role of biodosimetry—particularly cytogenetic markers—in understanding cumulative exposure and strengthening clinical protection strategies.

#### Day 3 (Wednesday): nuclear medicine applications and Fukushima case study

Dr. Takashi Kudo (Nagasaki University) opened Day 3 with an overview of radiation use in nuclear medicine, emphasizing that medical exposures now contribute the largest share of public radiation dose. He presented strategies for dose optimization in nuclear cardiology based on INCAPS best practices [36], including reducing thallium use [37], limiting dual-isotope protocols [38], weight-based dosing, and utilizing advanced SPECT/PET systems [39, 40]. These measures reduce radiation exposures for both patients and staff. Dr. Kudo also discussed emerging theranostics such as Ga-68/Lu-177 PSMA [41], highlighting the need for personalized dosimetry to optimize tumor targeting while minimizing toxicity to organs such as kidneys. Practical concerns—such as I-131 room contamination—underscored the importance of radiation hygiene and controlled clinical environments.

The participants visited Hiroshima University Hospital to observe the clinical workflows relevant to diagnostic imaging and radiation safety.

In the afternoon, Dr. Masatoshi Suzuki (Tohoku University) provided a comparative analysis of the Fukushima and Chernobyl nuclear accidents. He outlined radionuclide release characteristics, environmental contamination patterns, and pathways of internal exposure, focusing on Cs-137 and I-131. Practical measures for reducing internal contamination—food control, protective behavior, and environmental monitoring—were highlighted. He clarified distinctions between deterministic effects, which occur above thresholds, and stochastic risks such as cancer, reinforcing the importance of long-term dose management following nuclear accidents [42].

Dr. Yuko Nakamura (Hiroshima University) discussed the increasing global use of CT and its associated radiation risks. Japan’s high CT utilization was noted as a key public health consideration. She reviewed epidemiological evidence for elevated cancer risk—particularly in children [9, 43]—and explained the relevance of the Linear-Non-Threshold (LNT) model in estimating low-dose effects [44]. She emphasized justification and optimization as core principles of CT radiation protection, the role of Diagnostic Reference Levels [45], and the need to balance radiation reduction with maintenance of diagnostic image quality.

Day 3 provided participants with essential insights into nuclear medicine dose optimization, theranostic applications, CT radiation risks, and internal dose issues following nuclear accidents. The combination of lectures, hospital observation, and historical context strengthened participants’ understanding of both the technical and societal dimensions of radiation use and protection.

#### Day 4 (Thursday): environmental dose effects and chromosomal studies

Day 4 opened with Dr. Masatoshi Suzuki (Tohoku University), who presented an overview of ecological radiation assessment following the Fukushima Daiichi accident. He explained the selection of 12 Reference Animals and Plants (RAPs) as standardized indicators for evaluating biological impacts in contaminated ecosystems [46]. These species, chosen for ecological relevance and radiosensitivity, form the basis for assessing long-term environmental dose effects. Dr. Suzuki summarized post-accident monitoring results, including radiation levels in multiple environmental compartments and observed ecological changes [47]. He also outlined an ongoing Tohoku University project examining disaster-affected wildlife through integrated biological and radiological assessments to support evidence-based environmental recovery.

Dr. Ritsu Sakata (RERF) presented key findings from more than seven decades of epidemiological studies of atomic bomb survivors, focusing on the Life Span Study (LSS) and Adult Health Study (AHS) cohorts. These large, well-characterized cohorts enabled precise dose–response analyses for leukemia and solid cancers, demonstrating clear radiation-related increases in cancer risk modified by sex and age at exposure [48, 49]. Beyond cancer outcomes, Dr. Sakata discussed evidence for radiation effects on non-cancer diseases and outcomes following *in utero* exposure. While developmental effects were observed in individuals exposed prenatally to high doses, long-term follow-up has shown no significant increase in cancer incidence or mortality in the F1 generation [50]. She emphasized that these epidemiological findings provide an essential population-level context for interpreting biodosimetry results, and that the absence of detectable transgenerational effects is consistent with cytogenetic evidence reported from chromosomal studies. Together, these data continue to serve as a cornerstone for international radiation protection standards and risk assessment.

After a short discussion, the afternoon continued with a peer-presentation session in which each participant gave a six-minute overview of their laboratory’s biodosimetry research or operational capacity. Topics included cytogenetic assay improvements, new molecular biomarkers, and country-specific challenges in dose assessment. The subsequent Q&A session facilitated technical exchange and opportunities for future collaboration. The IAEA then conducted the workshop evaluation, gathering participant feedback on program content, logistics, and future training needs. The day concluded with a laboratory visit at Hiroshima University, highlighting cytogenetic and molecular facilities relevant to biodosimetry research.

Day 4 provided participants a cohesive understanding of environmental radiation impact assessment and long-term human health effects, complemented by peer knowledge exchange and hands-on exposure to biodosimetry laboratory practices.

#### Day 5 (Friday): contributions from RERF and future directions in radiation research

Day 5 opened with a keynote lecture by Dr. Kazunori Kodama, Executive Director of RERF and Secretary of HICARE, summarizing the major contributions of long-term studies of atomic bomb survivors to global radiation protection. He traced the evolution from the Atomic Bomb Casualty Commission (ABCC) to RERF and highlighted how large-scale cohorts of over 120 000 survivors have provided robust dose–response data for cancer and non-cancer diseases. These findings have shaped international risk models adopted by UNSCEAR, ICRP, and the IAEA. Ongoing analyses include long-term health outcomes in survivors and their F1 offspring, with particular emphasis on those exposed before age 20 [51].

Dr. Kodama then outlined HICARE’s international programs, including training initiatives for medical professionals and contributions to international projects such as the IAEA Chernobyl Project. He emphasized emerging research priorities—such as improved dose–response modeling, expanded use of biological samples to study mechanisms of radiation effects, and enhanced long-term health surveillance—to strengthen preparedness for future radiological events.

### Implications for research and practice

The HICARE/IAEA 2025 workshop highlighted how biological and internal dosimetry are evolving into essential components of modern radiation protection, clinical decision-making, and emergency response. Participants reviewed both classical and emerging tools for dose reconstruction. Classical cytogenetic assays—such as the dicentric chromosome assay, micronucleus assay, and translocation analysis—remain central due to their robustness and validated dose–response characteristics [52]. Complementing these, molecular biomarkers including γH2AX foci and transcriptomic signatures were emphasized for their rapid processing times and growing potential for high-throughput analysis [53–55].

Key discussions pointed to a shift from stand-alone laboratory techniques toward integrated, scalable systems capable of supporting population-level dose assessment during emergencies. This underscores future research priorities such as validating new biomarkers across diverse exposure conditions and improving assay automation. Clinically, biodosimetry is increasingly relevant for personalized radiotherapy planning and for identifying radiosensitive individuals using cytogenetic and molecular endpoints [56]. Expanding its use in occupational health—particularly for fluoroscopy-exposed medical staff—also presents opportunities for translational research and long-term risk mitigation [57].

Internal dosimetry sessions reinforced the importance of combining biokinetic modeling, in vivo measurements, and bioassay data for accurate dose reconstruction. Personalized organ-specific dose assessment, supported by ICRP respiratory and alimentary tract models, has vital applications in managing accidental radionuclide intake, medical misadministrations, and chronic occupational exposures [3]. Integrating biological endpoints with physical dosimetry provides a more complete assessment framework, particularly when monitoring data are scarce or delayed [58].

From a public health standpoint, long-term monitoring approaches—such as tracking persistent chromosomal translocations in A-bomb survivors or evaluating in-utero exposures—demonstrated how biodosimetry contributes to evidence-based risk communication and protective actions after radiological events [59, 60]. Ethical and societal considerations were also highlighted, emphasizing the need for transparency, culturally sensitive communication, and sustained engagement with affected populations [61, 62]. Collectively, the workshop reaffirmed biodosimetry as a cornerstone discipline with expanding relevance across clinical medicine, environmental protection, occupational safety, and emergency preparedness.

### Recommendations

Building on the workshop’s outcomes, several priorities were identified to strengthen global biodosimetry capacity:

#### Strengthen regional and international networks

Participants emphasized the value of coordinated networks for resource sharing, harmonized methodologies, and rapid mobilization during radiological events. A regional consortium would facilitate interlaboratory comparisons, shared training programs, and collaborative validation of new assays [63, 64].

#### Enhance standardization and quality assurance

Variability in scoring criteria, calibration curves, and sample handling continues to limit comparability across labs. Wider adoption of internationally recognized Standard Operating Procedures (SOPs), regular proficiency testing, and standardized reporting frameworks will improve reliability [1, 65]. Support from bodies such as the IAEA and WHO will be essential.

#### Expand education and digital training resources

The workshop format should be extended through online modules, virtual case studies, and remote coaching to ensure sustained competency, especially for institutions with limited training access [66, 67]. Training should also incorporate internal dosimetry, ethics, and communication skills to prepare practitioners for multidisciplinary roles.

#### Invest in technology and laboratory infrastructure

Automation technologies—including metaphase finders, high-resolution imaging, and integrated data systems—are crucial to reducing analysis time and supporting large-scale emergency response [68]. Funding mechanisms should prioritize upgrades and technology adoption, supported by demonstration projects that show real-world workflow integration [63, 69].

#### Establish mentorship and exchange programs

Short-term placements at experienced biodosimetry centers and reciprocal expert exchanges will strengthen regional capacity, encourage knowledge sharing, and accelerate adoption of best practices [70, 71].

#### Integrate biodosimetry into national emergency preparedness

Countries should explicitly define biodosimetry functions in emergency plans, coordinate with medical and civil protection agencies, and conduct regular drills incorporating real-time dose estimation scenarios [72]. Elevating biodosimetry from a research activity to an operational capability is essential for effective radiological emergency management.

## CONCLUSION

The HICARE/IAEA 2025 workshop reaffirmed the central role of biodosimetry in contemporary radiation protection, emphasizing its relevance across clinical practice, public health preparedness, occupational safety, and environmental monitoring. Through combined exposure to classical cytogenetic assays and emerging molecular and internal dosimetry methods, participants strengthened their capacity to perform accurate and context-appropriate dose assessment.

The workshop demonstrated that biodosimetry is advancing from isolated laboratory techniques toward integrated systems capable of supporting real-time assessment during radiological incidents and personalized decision-making in medical and occupational settings. The collaborative setting further promoted knowledge exchange and highlighted the need for continued harmonization of methods and quality standards across regions.

Looking ahead, sustained commitment to research innovation, workforce training, infrastructure development, and international cooperation will be essential to fully realize the potential of biodosimetry. These efforts will support stronger national and regional resilience to evolving radiological risks and ensure that biodosimetry remains a robust, operational tool for protecting public and environmental health.
